# Pulmonary Embolism With Multiple Active Right Ventricular Thrombi in Transit Managed Using Anticoagulation With and Without Inferior Vena Cava Filter: A Report of Two Oncology Patients

**DOI:** 10.7759/cureus.59536

**Published:** 2024-05-02

**Authors:** Waleed Abdalla, Yasir Almalki, Noof Alkharoosi, Ahmed Basuoni

**Affiliations:** 1 Cardiology, Sultan Qaboos Comprehensive Cancer Care and Research Center, Muscat, OMN

**Keywords:** intracardiac thrombus, pulmonary embolism, clot in transit, rv thrombus, thrombus in transit

## Abstract

Pulmonary embolism (PE) in the context of a right ventricular (RV) thrombus in transit is a special situation requiring a quick response that differs according to many factors. It is a rare but alarming finding. There is no clear guide to date that outlines a common pathway for treatment, as many factors play a role in determining the treatment plan. The mere presence of a thrombus in transit in the right atrium or right ventricle with a concomitant PE carries a higher risk of morbidity and mortality than PE alone. We will examine two cases presenting with PE with concomitant RV multiple thrombi and a background of cancer and diffuse bilateral deep vein thrombosis. One case was treated with anticoagulation alone, and the other with an inferior vena cava (IVC) filter in addition to anticoagulation. They both had a stable course despite their high risks and the frightening appearance of the multiple floating and attached thrombi seen in their echocardiography, some of which newly appeared after the second day of anticoagulation. The cases reflect the effectiveness of echocardiography for detecting and guiding treatment even after starting anticoagulation as well as the good outcome in such cases with anticoagulation alone when no massive PE occurs.

## Introduction

Thrombus in transit is a highly alarming finding in echocardiography that should be acted upon urgently. It involves a floating thrombus in the right heart chambers on the path of causing pulmonary embolism (PE). It is usually associated with significant morbidity and mortality, manifested as massive PE in 30% of cases. Overall mortality for a pulmonary embolus and an associated thrombus in transit is around 45% [1,2]. There is no standard approach to the management of thrombosis in transit or thrombi in the right ventricular (RV) in general, as management should be done case-by-case with multidisciplinary coordination.

We will present two cases treated successfully with anticoagulation despite their presentation with PE with the burden of a significant RV thrombosis in transit.

## Case presentation

Case 1

Our first case was a 58-year-old female with stage 2 right breast cancer in remission. She was known to have post-anthracycline (chemotherapy)-induced cardiomyopathy with a reported ejection fraction (EF) of 20%. She was not a known diabetic and had neither hypertension nor ischemic heart disease (IHD). She did have chronic gastritis.

The patient’s event started after having an episode of gastritis, after which she stopped all her heart failure (HF) medications. A few days later, she complained of severe epigastric pain and shortness of breath (SOB) and was treated in a local hospital for gastritis with HF exacerbation. Her response to HF treatment was minimal, increasingly becoming dyspneic, tachypneic, and hypoxic, with bilateral lower limb edema. She was transferred to our center.

On presentation to Sultan Qaboos Comprehensive Cancer Care and Research Center (SQCCCRC), her examination showed the following: respiratory rate of 24/min, blood pressure (BP) of 100/70, heart rate of 90/min, and oxygen saturation of 87% on room air, which increased to 95% with 2 liters of oxygen via nasal prongs. Her jugular venous pressure (JVP) was raised and chest auscultation showed reduced breath sounds with crepitations in the mid and lower zones bilaterally. Routine lab investigations included an echocardiography. She also had a computed tomography pulmonary arteriography (CTPA) with a differential diagnosis of heart failure and PE (considering her background of malignancy). The tests also aimed to exclude pericardial or pleural effusion (malignant or benign) as another possibility.

**Figure 1:**
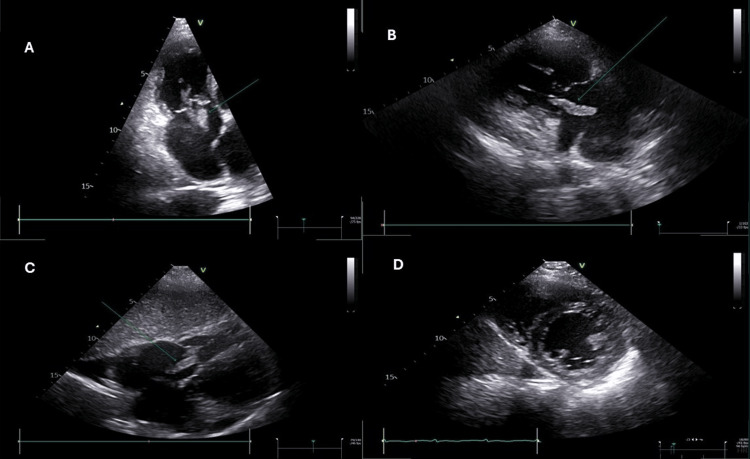
Case 1: The green arrow demonstrating a thrombus passing in and out the right ventricle (RV) across the tricuspid valve in panel A (the apical four-chamber RV dedicated view), panel B (the RV inflow view), and panel C (the subcostal view). Panel D shows the short axis view of papillary muscle level.

The results showed the following: electrocardiogram (ECG) showed normal sinus rhythm with T-wave inversion in the inferior, lateral, and V4-6 leads. Highly sensitive troponin T was 33 and N-terminal pro-brain natriuretic peptide (NT-ProBNP) was 13029 pg/ml. Echocardiography revealed a dilated left ventricle with severely reduced systolic function and severe global hypokinesia. EF was 23%, with restrictive diastolic function. The right ventricular systolic function was mildly reduced. There was moderate mitral regurgitation and severe tricuspid regurgitation (TR). Right ventricular systolic pressure (RVSP) was 50 mmHg. There was a clear RV thrombus in transit. Figure 1 and Video 1 from the first echocardiography of case 1 clearly demonstrated the RV thrombus passing in and out of the right ventricle across the tricuspid valve.

**Video 1:** Case 1: The green arrow shows a type 1 thrombus passing in and out of the right ventricle (RV) across the tricuspid valve (TV) in transit. Panel A shows the apical four-chamber right ventricular dedicated view, panel B shows the parasternal short axis view at the aortic valve level, panel C shows the subcostal view, and panel D shows the right ventricular inflow view.

**Figure 2:**
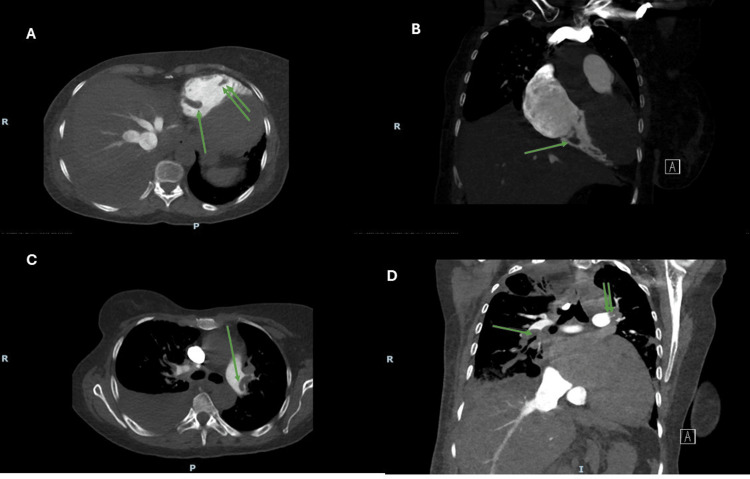
Case 1: Computed tomography pulmonary arteriography (CTPA). Panel A (axial view at the level of the right ventricle) showing the thrombus. Single green arrow and double green arrows pointing to the right ventricle thrombus in transit. Panel B (sagittal view) showing a thrombus in transit after passing through the tricuspid valve into the right ventricle. Panel C (axial view at the level of the pulmonary artery) showing a saddle-shaped embolus at the bifurcation of the left pulmonary artery and upper left lobe branch. Panel D (sagittal view at the level of pulmonary arteries). Single and double green arrows showing the continuation of the thrombi from the main pulmonary arteries into the lobar and inter-lobar branches bilaterally.

CTPA confirmed bilateral PE, There were moderate-sized, saddle-shaped filling defects in bilateral main pulmonary arteries and emboli were also noted in segmental and sub-segmental pulmonary arteries bilaterally, as demonstrated in Figure 2. Lower limb Doppler confirmed bilateral extensive deep vein thrombosis (DVT).

**Figure 3:**
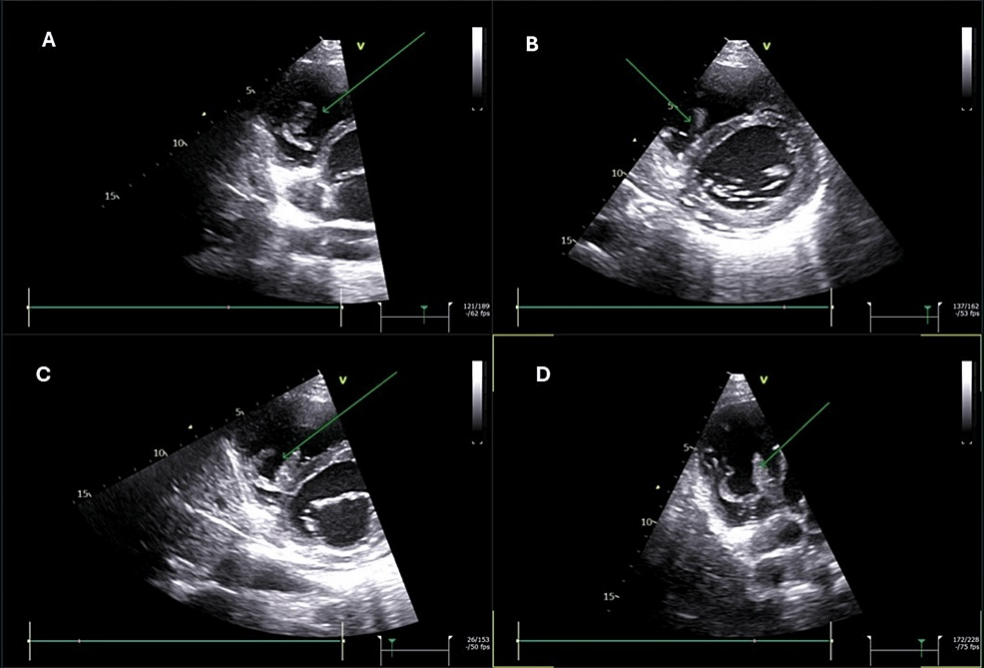
Case 1: Follow-up echocardiography. The green arrow shows a new thrombus curling in the right ventricle. Panel A: Short-axis view, mitral-valve level, zoomed-in view. Panel B: Short-axis view, papillary-muscle level. Panel C: Short-axis view, mitral-valve level. Panel D: Four-chamber dedicated right-ventricular view.

The patient was started on unfractionated heparin infusion since she was hemodynamically compensated, and there was no role for any invasive approach or thrombolysis. The option of thrombolysis (systemic or catheter-directed) was reserved in case of hemodynamic decompensation, together with the option of mechanical thrombectomy as a possible alternative to thrombolysis. Her follow-up echocardiography after two days showed two new larger mobile RV thrombi, demonstrating possible new thrombi in transit and increasing her risk of massive PE. Figure 3 and Video 2 are from the second (follow-up) echocardiography showing the new thrombus detected curled in the right ventricle after starting anticoagulation.

**Video 2:** Case 1: Follow-up echocardiography. It shows the new thrombus curling in the right ventricle in the parasternal short-axis view at the mitral-valve level.

Another step was taken by the pulmonology team: insertion of an inferior vena cava (IVC) filter to prevent these ongoing embolizing thrombi from her extensive bilateral DVT from causing massive PE (anticoagulation was ongoing).

On day five, a repeat echocardiography showed complete resolution of the large RV thrombi. On day seven, she was switched to oral anticoagulation.

The patient improved clinically, and all her symptoms improved. Her heart failure medications were upgraded and optimized. The IVC filter was removed after four weeks with no complications. She was clinically well compensated at discharge and is still being followed regularly.

Case 2

Our second patient was a 39-year-old female who was newly diagnosed with non-small-cell lung carcinoma (histology adenocarcinoma) and was referred to our center for further cancer management. She was also known to have a history of ablation for supraventricular tachycardia (SVT). She came to SQCCCRC for a regular appointment to start chemotherapy with carboplatin and pemetrexed (in a palliative setting). She was found to have a low BP of 80/70 and tachycardia but no SOB, so she was shifted to the intensive care unit (ICU) and was resuscitated. Blood investigations were taken, and she also had an echocardiography and lower limb Doppler. It is known in our practice that cancer patients have a very high thrombo-embolic risk, so our threshold to investigate for PE in cases of tachycardia with or even without SOB is low, especially when there is low blood pressure, so a CTPA was also requested. Her complete blood count showed neutrophilic leukocytosis, and her C-reactive protein was 126. The CTPA showed that she had PE in the right-side pulmonary artery and left-side segmental pulmonary artery, in addition to features of lymphangitis carcinomatosis.

The patient was immediately started on intravenous fluids and a therapeutic dose of enoxaparin and antibiotics were started after sending blood cultures. Thrombolysis was kept ready in case of clinical evidence of massive PE or no response to supportive measures. Echocardiography showed normal right and left ventricular systolic functions, mild TR, RVSP was 50 mmHg, no evidence of RV strain, and mild pericardial effusion. The lower limb Doppler showed bilateral extensive DVT.

**Figure 4:**
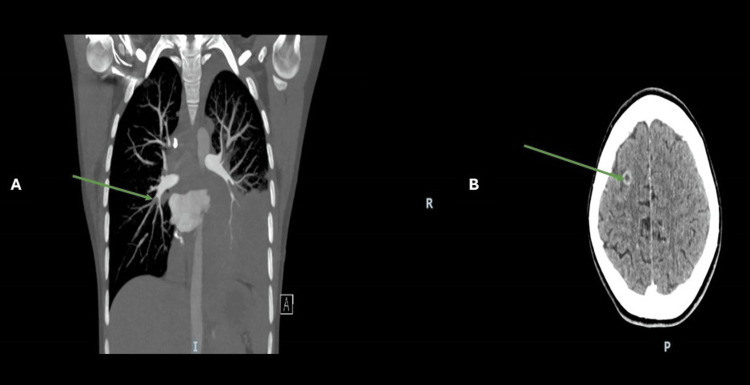
Case 2. Panel A, green arrow: Pulmonary embolism involving the right middle and right lower lobe segmental and subsegmental pulmonary arteries seen in CT pulmonary arteriography (CTPA). Panel B: Brain metastasis seen in head computed tomography. The green arrow demonstrates a 1 cm ring-enhancing intra-axial lesion at the level of the right frontal lobe suggestive of a metastatic lesion. It is surrounded by moderate vasogenic edema.

The patient’s condition improved, and her BP increased to acceptable readings. In the evening, she underwent computed tomography (CT) of the head, chest, pelvis, and cervical spine for the purpose of cancer staging to set her oncology treatment plan. The CT showed brain metastasis (frontal lobe); right vertebral vein thrombosis measuring about 12 mm; right gonadal vein thrombosis; and liver, kidney, and right adrenal metastasis. Figure 4 demonstrates the brain metastasis seen in the patient’s brain CT and the PE seen in CTPA.

**Figure 5:**
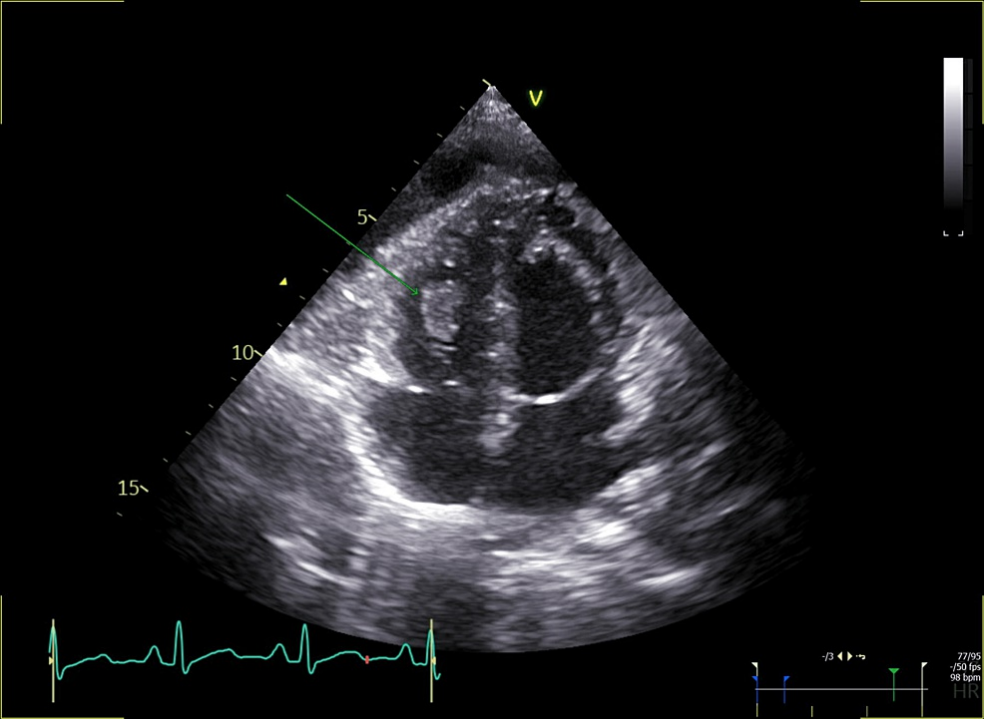
Case 2: Apical four-chamber view showing a thrombus in the right ventricle (green arrow).

The patient was stable and was started on cycle 1 of carboplatin and pemetrexed on day three. A follow-up echocardiography (on day three) showed a new thrombus detected in the right ventricle; the size was 1.6 cm x 0.9 cm, as demonstrated in Figure 5.

She was on day three of therapeutic enoxaparin. She was hemodynamically stable and, in light of her new brain metastasis, the decision was to continue anticoagulation only. On subsequent follow-up after three days, there was no evidence of RV thrombus, and the patient’s overall condition improved, allowing discharge a few days later. But she passed away a few weeks later from a complicated pneumonia.

## Discussion

Right heart thrombi in transit have a poor overall prognosis. The in-hospital mortality has been reported to be as high as 45% [2]. Patients treated with anticoagulation, thrombolytics, or surgical embolectomy differ in many aspects, making direct comparisons between the therapeutic strategies difficult [3]. Decision among potential therapies should carefully consider factors such as bleeding risks, hemodynamic state, malignancy, renal function, and RV function.

One comprehensive review of all cases of thrombus in transit from 1982 to 2022 made important observations. One was that type A thrombi have the highest risk of embolization. Another was that thrombi in transit were diagnosed when patients were having features of PE, and those who simultaneously had a right heart thrombus (RHT) in the setting of PE had three times as many poor short-term outcomes as those who had either alone [4,5].

In our first case, the type A thrombi were the type of thrombi embolizing the pulmonary arteries. Both of our cases had active thrombi in transit and simultaneous PE, and the short-term outcomes were good despite different medical approaches, i.e., anticoagulation alone in one case and the addition of an IVC filter in the other. These results support the fact that patients’ comorbidity and clinical presentation have more influence on outcome than do the type or character of the embolizing thrombi.

The medical approach we adopted was easy and effective. The challenge we faced in our cases was the thrombus fragmentation from the massive DVT leading to repeated embolization after starting anticoagulation; we know anticoagulation only prevents clot proliferation and does not dissolve already formed thrombi. That was more evident in our first case. There is no clear guideline for using IVC filters in patients having DVT actively showering thrombi in the setting of RV thrombi in transit after the early hours or days of starting anticoagulation. Nonetheless, we used an IVC filter to prevent massive PE because of the ongoing thrombi showering, thereby opening a window for anticoagulation to take full effect. A study performed on patients having right heart thrombi and PE, where the patients were given anticoagulation or anticoagulation with reperfusion, concluded that there was no significant statistical difference in all-cause mortality and major bleeding [6]. But because our patients had cancer with metastasis and were stable hemodynamically, we preferred not to use any thrombolytic therapy unless obstructive PE occurred, and in that situation, the priority would have been for mechanical thrombectomy, which is another suitable alternative with lesser bleeding risks but would require shifting the patient to another hospital.

Khosla et al. created a framework to approach patients with an RHT and concomitant PE. They considered the RHT as a marker of severity of disease and therefore suggested a lower threshold to use a more invasive and advanced approach in addition to anticoagulation. They set an exception to this for patients with small RHT, small pulmonary embolisms, and preserved RV function, in addition to patients considered to have a low life expectancy [7]. When applying that framework to our cases, we note that our patients had relatively large RHT, and in one case, there were multiple right heart thrombi, but on the other hand, the RV size and function were preserved despite multiple pulmonary thrombi. Both cases responded well with anticoagulation, which may reflect the higher weight of the preserved RV function over the mere presence or character of the RHT.

The type of RV thrombus may be one of the factors that dictate the approach, especially if it is unattached and highly mobile, in addition to the other features of thrombi that predict the potential to embolize and cause abrupt RV decompensation and shock may lead to a more aggressive approach, but in our cases, the patients’ clinical presentations and hemodynamic status influenced the outcome more than did the thrombus numbers, characters, and sizes.

## Conclusions

Anticoagulation alone is an efficient and acceptable approach in patients where thrombolysis or surgery are not required or are contraindicated, especially in the elderly population, patients with advanced cancer, and those with a high risk of bleeding or stroke. All cases of PE with a detected RV thrombus in transit should be discussed in a team that includes all the specialties involved in the care of these patients, including pulmonology, cardiology, ICU, interventional, and cardiothoracic surgery teams. One useful tool to use is echocardiography, which should be used daily in selected cases with significant DVT, even after starting anticoagulation in hemodynamically stable patients, as migrating thrombi from DVT may start appearing after day one or two, leading to new thrombus embolization. In that case, if the patient is still compensated hemodynamically, an IVC filter can be inserted, buying time for anticoagulation to take full effect.

The outcome in patients with PE and RV thrombus in transit depends on the clinical presentation and course of action rather than on identifying the thrombus in transit or its type alone. The management of this subset of patients remains difficult, considering the scarcity of data on this subject.
